# Combined Anterior–Posterior vs. Posterior-Only Approach in Adult Spinal Deformity Surgery: Which Strategy Is Superior?

**DOI:** 10.3390/jcm13030682

**Published:** 2024-01-24

**Authors:** Iyan Younus, Hani Chanbour, Jeffrey W. Chen, Graham W. Johnson, Tyler Metcalf, Alexander T. Lyons, Soren Jonzzon, Campbell Liles, Steven G. Roth, Amir M. Abtahi, Byron F. Stephens, Scott L. Zuckerman

**Affiliations:** 1 Department of Neurological Surgery, Vanderbilt University Medical Center, Nashville, TN 37212, USA; iyan.younus@vumc.org (I.Y.); hani.chanbour@vumc.org (H.C.); soren.jonzzon@vumc.org (S.J.); david.c.liles.1@vumc.org (C.L.); sgr2141@cumc.columbia.edu (S.G.R.); amir.m.abtahi@vumc.org (A.M.A.); byron.stephens@vumc.org (B.F.S.); 2 Department of Neurological Surgery, Baylor College of Medicine, Houston, TX 77030, USA; jwchen.rj@gmail.com; 3 School of Medicine, Vanderbilt University, Nashville, TN 37232, USA; graham.w.johnson@vanderbilt.edu (G.W.J.); metc11@osumc.edu (T.M.); alexander.t.lyons@vanderbilt.edu (A.T.L.); 4 Department of Orthopedic Surgery, Vanderbilt University Medical Center, Nashville, TN 37232, USA

**Keywords:** adult spinal deformity, anterior–posterior, posterior only, approach, sagittal malalignment, outcomes

## Abstract

**Introduction:** Whether a combined anterior–posterior (AP) approach offers additional benefits over the posterior-only (P) approach in adult spinal deformity (ASD) surgery remains unknown. In a cohort of patients undergoing ASD surgery, we compared the combined AP vs. the P-only approach in: (1) preoperative/perioperative variables, (2) radiographic measurements, and (3) postoperative outcomes. **Methods:** A single-institution, retrospective cohort study was performed for patients undergoing ASD surgery from 2009 to 2021. Inclusion criteria were ≥5-level fusion, sagittal/coronal deformity, and 2-year follow-up. The primary exposure was the operative approach: a combined AP approach or P alone. Postoperative outcomes included mechanical complications, reoperation, and minimal clinically important difference (MCID), defined as 30% of patient-reported outcome measures (PROMs). Multivariable linear regression was controlled for age, BMI, and previous fusion. **Results:** Among 238 patients undergoing ASD surgery, 34 (14.3%) patients underwent the AP approach and 204 (85.7%) underwent the P-only approach. The AP group consisted mostly of anterior lumbar interbody fusion (ALIF) at L5/S1 (73.5%) and/or L4/L5 (38.0%). Preoperatively, the AP group had more previous fusions (64.7% vs. 28.9%, *p* < 0.001), higher pelvic tilt (PT) (29.6 ± 11.6° vs. 24.6 ± 11.4°, *p* = 0.037), higher T1 pelvic angle (T1PA) (31.8 ± 12.7° vs. 24.0 ± 13.9°, *p* = 0.003), less L1-S1 lordosis (−14.7 ± 28.4° vs. −24.3 ± 33.4°, *p* < 0.039), less L4-S1 lordosis (−25.4 ± 14.7° vs. 31.6 ± 15.5°, *p* = 0.042), and higher sagittal vertical axis (SVA) (102.6 ± 51.9 vs. 66.4 ± 71.2 mm, *p* = 0.005). Perioperatively, the AP approach had longer operative time (553.9 ± 177.4 vs. 397.4 ± 129.0 min, *p* < 0.001), more interbodies placed (100% vs. 17.6%, *p* < 0.001), and longer length of stay (8.4 ± 10.7 vs. 7.0 ± 9.6 days, *p* = 0.026). Radiographically, the AP group had more improvement in T1PA (13.4 ± 8.7° vs. 9.5 ± 8.6°, *p* = 0.005), L1-S1 lordosis (−14.3 ± 25.6° vs. −3.2 ± 20.2°, *p* < 0.001), L4-S1 lordosis (−4.7 ± 16.4° vs. 3.2 ± 13.7°, *p* = 0.008), and SVA (65.3 ± 44.8 vs. 44.8 ± 47.7 mm, *p* = 0.007). These outcomes remained statistically significant in the multivariable analysis controlling for age, BMI, and previous fusion. Postoperatively, no significant differences were found in mechanical complications, reoperations, or MCID of PROMs. **Conclusions:** Preoperatively, patients undergoing the combined anterior–posterior approach had higher PT, T1PA, and SVA and lower L1-S1 and L4-S1 lordosis than the posterior-only approach. Despite increased operative time and length of stay, the anterior–posterior approach provided greater sagittal correction without any difference in mechanical complications or PROMs.

## 1. Introduction

Adult spinal deformity (ASD) surgery requires complex operations to improve spinal alignment, which can lead to major improvements in quality of life; however, perioperative morbidity remains high [1,2]. Despite durable improvement in quality of life after ASD surgery, major variation exists in surgical technique. Various approaches to ASD surgery include the anterior, trans-psoas, pre-psoas, or traditional posterior approaches [3,4,5]. Minimally invasive techniques (MIS), mainly tubular work and percutaneous screw placement, have also become popular [6]. The two most popular approaches are the staged combined anterior–posterior (AP) approach and the traditional posterior-only (P) approach. The AP and P approaches have been compared in a small number of studies, with most data extrapolated from comparing transforaminal or posterolateral interbody fusion (TLIF/PLIF) to anterior lumbar interbody fusion (ALIF) [7,8,9,10,11].

The anterior approach in ASD surgery has been reported to have several mechanical advantages, mainly allowing a large, lordotic implant to be placed at low lumbar levels [4]. Moreover, the anterior approach allows for direct removal of anterior osteophytes that can help realign the lumbosacral fractional curve and indirectly decompress the low lumbar nerve roots. Furthermore, the anterior approach allows for the insertion of larger and more lordotic interbody grafts, allowing for increased surface area for fusion and the placement of bone grafts under compressive forces to boost fusion potential, which has been reported to decrease the rate of posterior implant loosening and failure [10].

There has been a steady rise in the number of reported combined AP approaches for ASD surgery [7,8,9,10,11]. Several factors, such as high complication rates with aggressive posterior column corrections and the availability of novel anterior implants, have contributed to this trend [3]. However, there is still a paucity of long-term data on the clinical, mechanical, and radiographic outcomes of the AP approach compared with the P-only approach. Therefore, we sought to compare the AP approach with the P-only approach for (1) preoperative/perioperative variables, (2) radiographic measurements, and (3) and postoperative outcomes.

## 2. Materials and Methods

### 2.1. Study Design

A retrospective, single-institution study of patients undergoing ASD surgery with at least 2-year follow-up was conducted from 2009 to 2021. A total of 5 fellowship-trained neurosurgery and orthopedic spine surgeons contributed to this registry. Postoperative patient-reported outcome measures (PROMs) were collected by five full-time research personnel. Institutional Review Board (IRB) approval was obtained (IRB#220894).

### 2.2. Patient Selection

All patients included in the study were ≥18 years old and underwent elective surgery for ASD. Inclusion criteria were: 5-level fusions or more, Cobb angle ≥ 30°, sagittal vertical axis (SVA) ≥ 5 cm, coronal vertical axis (CVA) ≥ 3 cm, pelvic tilt (PT) ≥ 25°, or thoracic kyphosis (TK) ≥ 60°. Patients were required to have a minimum of 2-year follow-up. Patients received either a combined AP approach within the same anesthetic setting or staged within 1–2 days or a P-only approach, most often performed within the same anesthetic setting. The decision for a combined AP or P-only approach was typically based on the surgeon’s discretion, taking into consideration specific patient factors and the desired surgical goals. Generally, when a more extensive correction of sagittal alignment was needed, surgeons may have opted for the AP approach to achieve better correction.

### 2.3. Outcome Variables

Patient demographic and clinical data, including age, sex, body mass index (BMI), smoking status, and comorbidities (none, one, ≥two), were collected. Intraoperative measures, such as total instrumented levels, location of upper instrumented vertebra (UIV), number of interbody fusions, primary surgeon, blood loss as measured by estimated blood loss (EBL), calculated blood loss, and perioperative hemoglobin, were also collected.

Postoperative location of discharge and length of stay were documented. Complications, such as proximal/distal junctional kyphosis (PJK/DJK), rod fracture, and pseudoarthrosis, were recorded. The study examined patients who developed PJK. PJK was defined as an angle between the UIV inferior endplate and UIV + 2 superior endplate ≥ 10° and a concomitant ≥ 10° change compared to preoperative imaging [12]. PJF was defined as further progression on the PJK spectrum to include vertebral fracture of UIV or UIV + 1, subluxation between UIV and UIV + 1, failure of fixation, and/or neurological deficit [13,14].

Two-year PROMs, including Oswestry Disability Index (ODI), EuroQoL Group (EQ-5D), and numeric rating scale (NRS) for back and leg pain (NRS-BP and NRS-LP, respectively) were collected [15,16]. The minimally clinically important difference (MCID) was calculated for ODI, EQ-5D, and NRS [17,18].

Preoperative and postoperative radiographic measures were obtained, including coronal measurement of C7-PL and major Cobb, sagittal measures of L1-L4, lumbar lordosis (LL), pelvic incidence (PI), sacral slope (SS), sagittal vertical axis (SVA), pelvic tilt (PT), T1 pelvic angle (TPA), and L1-S1 angle. Variations in sagittal alignment were classified according to the Roussouly classification [19]. Clinically meaningful pelvic retroversion was defined as a PT > 50% of the PI.

### 2.4. Surgical Technique

Anterior surgery in the combined AP cohort primarily involved ALIF at L5/S1 or L4/5 or both. After exposure of the anterior part of the disc, the anterior longitudinal ligament was transversely incised, and the disc was completely removed. Next, the vertebral endplates were cleared of cartilage using sharp curettes, taking care that damage to the subchondral bone of the endplates was avoided. Maximum distraction of disc space was achieved by manual lordotic force. After a satisfactory trial implantation, the ALIF cage was filled with a morselized cancellous allograft and implanted. Bone morphogenic protein-2 (BMP-2) was used in almost all ALIFs but was left to the surgeon’s discretion.

Posterior surgery in the combined AP group and the P-only group involved instrumentation via an open posterior approach. Subperiosteal exposure of the dorsal spine using a standard midline approach, adequate decompression, pedicle screw instrumentation, and with or without a TLIF was performed. The TLIF was performed in the standard technique as has been described, and use of a bullet or banana cage was left to the surgeon’s discretion [20].

### 2.5. Statistical Analysis

Descriptive statistics were used to compare ASD patients who received combined AP vs. P-only approach for surgical correction. Continuous variables were reported as means and standard deviations, while categorical variables were reported as frequencies. To evaluate normal distribution and variance for continuous variables, the Shapiro–Wilk test and F-test were employed, respectively. Histograms were a qualitative assessment of normality. Normally distributed data with equal variance were analyzed using a two-tailed *t*-test, while nonparametric data were compared with the Wilcoxon signed-rank or Mann–Whitney test. For nominal data, χ^2^ or Fisher’s exact test was utilized in smaller samples. Univariate and multivariable analyses were conducted, controlling for patient age, BMI, and previous fusion. A significance level of *p*-value < 0.05 was considered statistically significant. All analyses were performed using R version 4.2.1 (The R Foundation, Vienna, Austria).

## 3. Results

### 3.1. Patient Demographics and Preoperative Data

**Figure 1:**
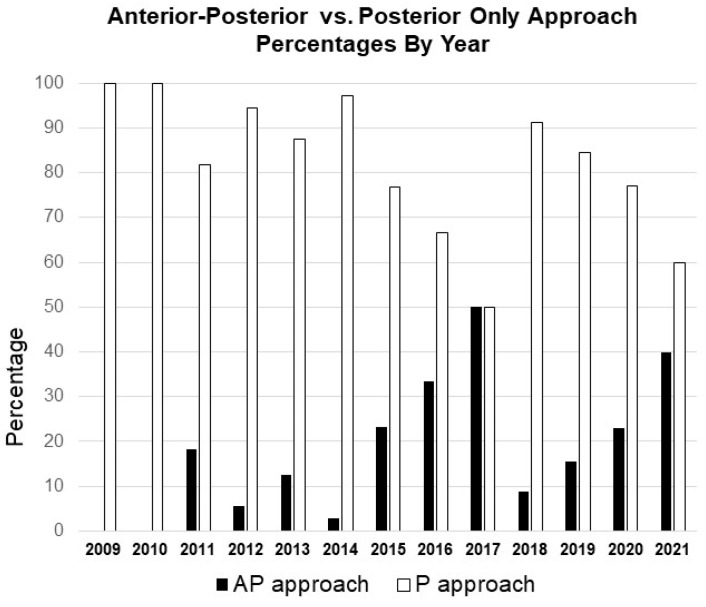
Bar graph comparing the rate of the combined AP vs. P-only approaches by year.

**Figure 2:**
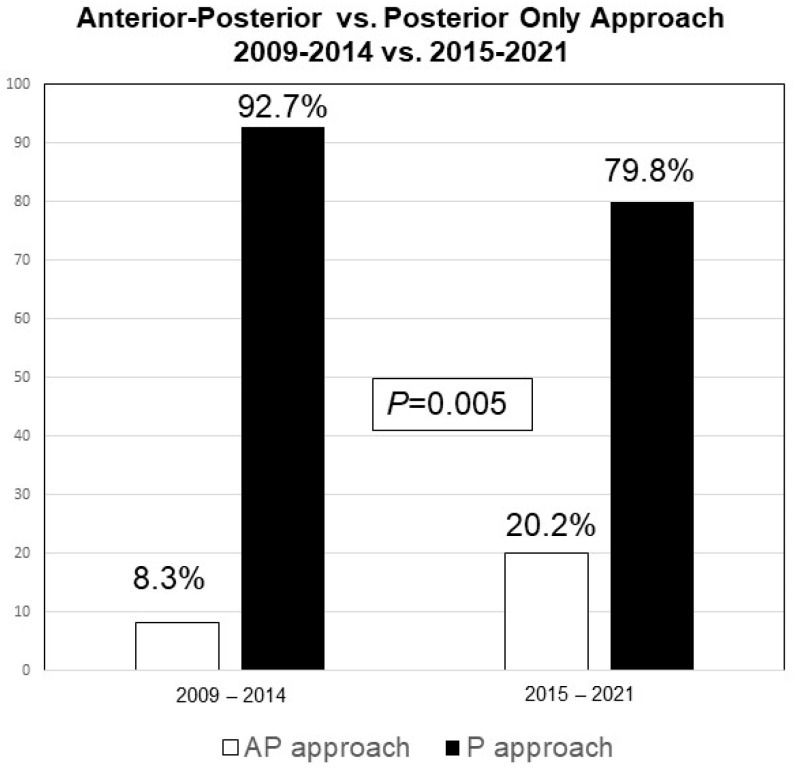
Bar graph comparing the rate of the combined AP vs. P-only approaches between 2009–2014 and 2014–2021.

Among the 238 patients undergoing ASD surgery, 34 (14.3%) patients underwent the combined AP approach, and the remaining 204 (85.7%) patients underwent the P-only approach (Table 1). The progression of the AP approach throughout the years is depicted in Figure 1 and Figure 2.

Patients who underwent the AP approach were slightly younger (62.8 ± 9.8 vs. 63.5 ± 18.4years, *p* = 0.048) and had higher BMI (31.2 ± 7.6 vs. 28.5 ± 6.8, *p* = 0.026) compared to patients in the P-only approach. Aside from age and BMI, the two cohorts shared similar demographics with regards to sex (*p* = 0.951), race (*p* = 0.130), and comorbidities (*p* = 0.269).

Patients in the AP group were significantly more likely to have received prior fusions (64.7% vs. 28.9%, *p* < 0.001), which mainly consisted of < 5-level fusion, and the indications were predominantly associated with degenerative conditions. Radiographically, the AP group had higher preoperative PT (29.6 ± 11.6° vs. 24.6 ± 11.4°, *p* = 0.037), higher T1PA (31.8 ± 12.7° vs. 24.0 ± 13.9°, *p* = 0.003), less L1-S1 lordosis (−14.7 ± 28.4° vs. −24.3 ± 33.4°, *p* < 0.039), less L4-S1 lordosis (−25.4 ± 14.7° vs. 31.6 ± 15.5°, *p* = 0.042), and higher SVA (102.6 ± 51.9 vs. 66.4 ± 71.2 mm, *p* = 0.005). In both cohorts, the most common lordotic apex was at L5 (32.4% vs. 34.8%, *p* = 0.357), and the most common Roussouly classification was Type 2 (47.1% vs. 40.3%, *p* = 0.531). There was, however, a significantly higher number of patients within the combined AP cohort with clinically meaningful pelvic retroversion with a PT > 50% of PI (64.7% vs. 41.6%, *p* = 0.012).

### 3.2. The Combined Anterior–Posterior Group

**Figure 3:**
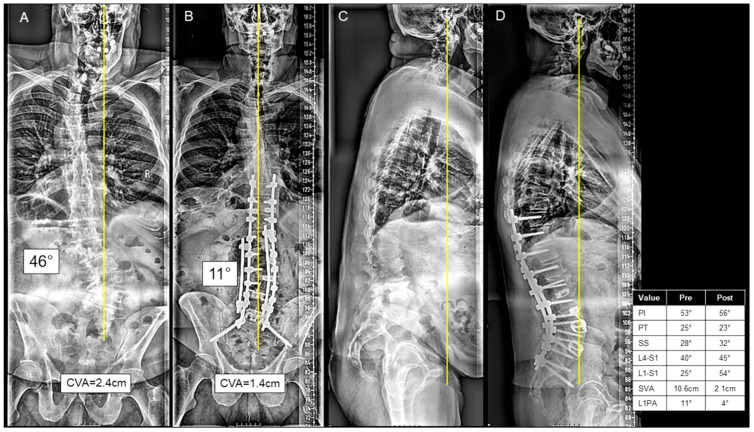
(**A**–**D**) A case presentation of a 67-year-old male presenting with L3-L4 left-sided radiculopathy and sagittal malalignment due to lumbar kyphosis causing severe mechanical back pain on the postero-anterior (PA) (**A**) and lateral X-rays (**C**). The patient underwent a stage 1 anterior lumbar interbody fusion at L4-5 and L5-S1. Two days later, the patient underwent a posterior approach consisting of T10-ilium instrumentation, posterior column osteotomies from T12-L5, inferior facetectomies from T10-S1, and L2/L3 and L3/L4 transforaminal lumbar interbody fusion, as seen on the postoperative PA (**B**) and lateral X-rays (**D**). The patient was discharged to IPR at postoperative day 9.

For patients in the AP group, the anterior approach consisted mostly of ALIF at L5/S1 (73.5%), L4/L5 (38.0%), L3/L4 (17.6%), L2/L3 (17.6%), and L1/L2 (2.9%). At L5/S1, the mean height of the implant was 10.9 ± 3.0 mm, and mean lordosis was −23.2 ± 32.5°. At L4/5, the mean height of the implant was 10.2 ± 3.0 mm, and mean lordosis was −10.7 ± 16.2°. Half of the AP cases were staged 18 (52.9%), separated by a mean of 2.1 (range 1–5) days. A three-column osteotomy was performed in 10 (29.4%) cases. A thoracolumbar UIV to sacrum/pelvis fusion was performed in 16 (47.0%) cases, and an upper/middle thoracic to sacrum/pelvis fusion was performed in 15 (44.1%) cases. A representative combined AP case is presented in Figure 3A–D.

### 3.3. The Posterior Only Group

**Figure 4:**
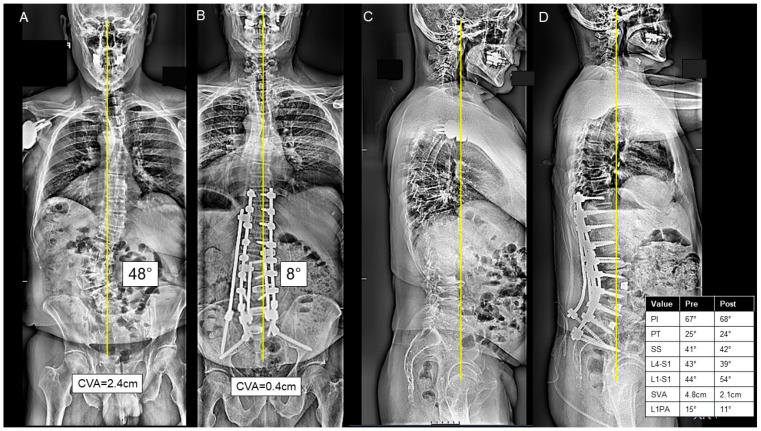
(**A**–**D**) A case presentation of a 67-year-old male with severe L2 and L3 right-sided radiculopathy, left-sided L4 radiculopathy, severe mechanical back pain, and inability to walk for more than 5–10 min, who was found to have a significant degenerative scoliosis in the thoracolumbar and lumbar spine on the postero-anterior (PA) (**A**) and lateral X-rays (**C**). The patient underwent a posterior-only approach consisting of T10-ilium posterior spinal instrumentation; T10-S1 inferior facetectomies; L1-S1 posterior column osteotomies; a total of 3 transforaminal lumbar interbody fusions at L3/L4, L4/L5, L5/S1; decompressive foraminotomies at L5/S1, L4/L5, L3/L4 and L2/L3; and a cobalt kickstand rod from T11/12-S1 with compression and distraction forces to correct the scoliosis, as seen on the postoperative PA (**B**) and lateral X-rays **(D**). The patient was discharged home at postoperative day 5.

For the P-only group, the posterior approach included lumbar interbodies in 36 (17.6%) patients. These were most commonly L4-L5 (36.1%), L5-S1 (58.3%), L3-L4 (25.0%), and L2-L3 (11.1%). The majority of P-only cases were performed in one anesthetic setting. A three-column osteotomy was performed in 34 (16.7%) cases. A thoracolumbar UIV to sacrum/pelvis fusion was performed in 58 (28.4%) cases, and an upper/middle thoracic to sacrum/pelvis fusion was performed in 103 (50.4%) cases. A representative P-only case is presented in Figure 4A–D.

### 3.4. Perioperative Outcomes

Perioperative variables are summarized in Table 2. The AP approach had longer operative time (553.9 ± 177.4 vs. 397.4 ± 129.0 min, *p* < 0.001), longer hospital length of stay (8.4 ± 10.7 vs. 7.0 ± 9.6 days, *p* = 0.026), and more interbodies placed (100% vs. 17.6%, *p* < 0.001). The AP group also had more mean total instrumented levels (11.4 ± 3.3 vs. 10.4 ± 3.1 levels), but this was not significantly different (*p* = 0.065). Both groups had similar rates of EBL (1285.1 ± 1147.3 vs. 1480.7 ± 1246.8 mL, *p* = 0.194) and discharge home vs. to other locations (*p* = 0.967). As a sub-analysis, we attempted to look at LOS in the AP group after the second stage to see if LOS was more similar when taking out time from the anterior stage. Comparing the LOS from the second stage of the AP group to the total LOS in the P-only group, no significant difference was seen (4.5 ± 1.6 vs. 6.9 ± 9.6 days, *p* = 0.296).

### 3.5. Radiographic Outcomes

No significant differences were found in postoperative radiographic parameters between the two groups (Table 3. However, regarding change in alignment, the combined AP group had a greater T1PA correction (13.4 ± 8.7° vs. 9.5 ± 8.6°, *p* = 0.005), greater improvement in L1-S1 lordosis (−14.3 ± 25.6° vs. −3.2 ± 20.2°, *p* < 0.001) and L4-S1 lordosis (−4.7 ± 16.4° vs. 3.2 ± 13.7°, *p* = 0.008), and a greater degree of SVA correction (65.3 ± 44.8 vs. 44.8 ± 47.7 mm, *p* = 0.007). These outcomes remained statistically significant in multivariable analysis controlling for age, BMI, and previous fusion (Table 4). 

Preoperatively, 23 (67.6%) patients had clinically significant pelvic retroversion in the AP group compared to 105 (52.0%) in the P-only group (*p* = 0.097). The mean PT in the AP group vs. P-only group was 29.6 ± 11.6 vs. 24.6 ± 11.4, respectively (0.037). Postoperatively, 14 (41.2%) patients had clinically meaningful pelvic retroversion in the AP group compared to 81 (39.7%) in the P-only group (*p* > 0.999).

Interestingly, patients undergoing the AP approach were significantly more likely to have pelvic tilt improvement by more than 5° compared to the P-only approach (50.0% vs. 27.5, *p* = 0.043). The association between the AP approach and improved pelvic tilt was also seen in the multivariable logistic regression controlling for the aforementioned variables (OR = 2.22, 95%CI = 1.01–4.95, *p* = 0.049).

### 3.6. Clinical and Patient-Reported Outcome Measures

Clinical measures and PROMs are summarized in Table 5. At 2-year follow-up, there were no significant differences in rates of pseudarthrosis (20.6% vs. 28.9%, *p* = 0.315), rod fracture (14.7% vs. 20.1%, *p* = 0.461), PJK (54.5% vs. 46.7%, *p* = 0.404), DJK (2.9% vs. 3.4%, *p* > 0.999), or reoperation rates for any reason (35.3% vs. 37.7%, *p* = 0.784). Similar non-significant findings were observed in the multivariate regression analysis.

With regards to PROMs at 2 years, patients in the AP group reported higher ODI (42.5 ± 19.2 vs. 34.7 ± 19.4, *p* = 0.208), NRS-BP (5.9 ± 2.8 vs. 4.8 ± 2.9 *p* = 0.202), NRS-LP (3.5 ± 3.7 vs. 3.0 ± 3.2 *p* = 0.601), and lower EQ-5D (0.6 ± 0.2 vs. 0.7 ± 0.2 *p* = 0.332), but these were not significantly different. There was also no significant difference between the AP and P groups, respectively, in rates of achieving the MCID for ODI (38.5% vs. 56.0%, *p* = 0.233), NRS-BP (38.5% vs. 53.0%, *p* = 0.324), NRS-LP (53.8% vs. 67.4%, *p* = 0.336), and EQ-5D (15.4% vs. 8.0%, *p* = 0.323).

## 4. Discussion

The present study evaluated outcomes in patients undergoing the combined AP vs. P-only approaches for ASD surgery. Patients undergoing the combined AP approach had greater preoperative sagittal malalignment with significantly higher preoperative PT and T1PA and lower L1-S1 and L4-S1 lordosis. Postoperatively, the combined AP approach provided better sagittal correction due to significantly greater change in postoperative T1PA correction, L1-S1 lordosis change, L4-S1 lordosis change, and SVA correction. There was no significant difference in mechanical complications or PROMs between the AP and P-only approaches. These findings support that the AP approach was chosen in cases of greater sagittal malalignment and, postoperatively, provided greater sagittal correction, with a similar profile of mechanical complications and PROMs.

The AP group had more sagittal malalignment preoperatively and also exhibited greater postoperative sagittal correction than the P-only group, specifically in the parameters of T1PA, L1-S1, L4-S1, and SVA. The powerful ability of the anterior approach to restore low lumbar lordosis has been well documented [7,21]. Haddad et al. [7] found a better SVA, PT, relative lumbar lordosis, and relative pelvic version in patients undergoing the AP vs. P-only approach. Similarly, Stephan et al. [21] reported a 5–15° segmental lordosis gain in the AP approach compared to the P-only approach at L4-L5 and L5-S1. Not only did our results corroborate these findings, but we also showed that rates of ALIFs being performed increased over time. As the field of ASD has evolved, surgeons have gained new appreciation for the importance of L4-S1 lordosis, evidenced by Roussouly and the Global Alignment and Proportion (GAP) score, and at our institution, our surgical approach has followed suit [19,22,23]. During an ALIF procedure, a larger interbody can typically be inserted, which has potentially far more lordosis correction compared to a posteriorly placed interbody, where additional carpentry and osteotomies are needed.

Despite the potent ability of an ALIF to improve low lumbar sagittal alignment, these results showed that the combined AP approach had a significantly longer operative time and LOS but were discharged home at similar rates. These findings are consistent with a study by Haddad et al. [7], who reported that combined approaches had significantly longer surgeries (548 vs. 283 min) and needed longer ICU stays (74 vs. 27 h). However, they had comparable complication rates and significantly fewer readmissions (9.1% vs. 38.1%) and reoperations (18.2% vs. 43.2%) at 2 years. Theologis et al. [11] compared patients undergoing multiple-level lateral interbody fusions at the apex of the coronal deformity with posterior fixation to a matched cohort of adult deformity patients treated with the posterior-only approach. All patients had an L5/S1 interbody fusion. Similar to our study, patients in the combined group had significantly more levels fused, longer operative times, and longer lengths of stay. Haddad et al. [7] also reported that patients undergoing the combined approach appeared to require more aggressive surgical intervention due to the inherent nature of their deformities, leading to an increased rate of fixation to the pelvis and a tendency toward more instrumented levels. In our cohort, we also saw that patients undergoing the combined AP approach had significantly more interbodies placed and more total instrumented levels. Our postoperative radiographic outcomes are similar to a report by Ming-Kai et al. [8] in which patients undergoing the combined approach with ALIF significantly improved sagittal and coronal correction compared to the posterior-only cohort. Theologis et al. [11] also found that the combined group had significant improvement in postoperative radiographic parameters such as PT, LL, and PI-LL.

The current study demonstrated no significant difference in mechanical complications or PROMs, signifying that either approach can lead to good outcomes. Patients also had no significant difference in MCID for postoperative PROMs such as ODI, NRS-BP, NR-LP, and EQ-5D. Similarly, Leveque et al. and Mundis et al. [24,25] compared patients undergoing anterior column realignment (ACR) with lateral graft placement and sectioning of the anterior longitudinal ligament to posterior-only approaches and found similar clinical outcomes and similar rates of overall major complication. These two studies found no significant clinical differences other than in blood loss, which was significantly less in the ACR group compared to the posterior-only group. In comparison, Haddad et al. [7] reported that patients in the combined group had better clinical outcomes as measured by COMI and SRS-22, which can possibly be explained by their significantly lower readmissions and reoperations in the combined approach. Bae et al. [26] compared patients with a combined posterior approach with lateral lumbar interbody fusion (LLIF), and ALIF and posterior-only approaches and found lower rates of PJK and mechanical failure at the UIV and better ODI and SRS-22 scores in the posterior LLIF group only. Theologis et al. [11] found a significant improvement in ODI and visual analogue scale scores in patients undergoing a combined approach for ASD. Unlike our series, these advantages came at the expense of having significantly more major complications (56% vs. 13%) and postoperative leg weakness (31% vs. 6%) in the combined approach. Overall, our results show that regardless of the AP or P-only approach, similar mechanical complications and PROMs were seen.

### Limitations

This study has several limitations warranting discussion. First, the AP cohort had a significantly higher rate of prior fusion, which could have influenced surgical approach and need for greater sagittal correction. Therefore, we accounted for prior fusion in our multivariable analysis and still found a significantly greater improvement in postoperative sagittal correction with the AP approach. Third, this is a retrospective, single-institution, multi-surgeon study and these findings may have limited generalizability. Fourth, determining the precise onset of ASD poses a significant challenge, as patients typically seek medical attention when symptoms become severe. Fifth, while we controlled for age, BMI, and previous fusion in our analysis, it was challenging to incorporate further covariates due to the small sample size. Lastly, the retrospective nature of our study posed added challenges to precisely determine the reasons behind the surgeon’s chosen procedure. Future, prospective trials are encouraged to better control for these factors and elucidate the decision-making process regarding the surgical approach for each surgeon.

## 5. Conclusions

In patients undergoing ASD surgery, a combined anterior–posterior approach had higher preoperative rates of prior fusion, higher T1PA, lower L1-S1 and L4-S1 lordosis, and a higher PT compared to the posterior-only approach. Perioperatively, the combined anterior–posterior approach had increased operative time and length of stay but provided a better sagittal alignment correction without any difference in mechanical complications or PROMs.

## Figures and Tables

**Table 1:** Demographic, preoperative, and radiographic variables.

Variables	Total Cohort = 238	Combined N = 34	Posterior Only N = 204	*p*-Value
**Preoperative**				
Age, mean ± SD	63.4 ± 17.4	62.8 ± 9.8	63.5 ± 18.4	0.048
Female, *n* (%)	181 (76.1%)	26 (76.5%)	155 (76.0%)	0.951
BMI, mean ± SD	28.9 ± 7.0	28.3 ± 7.6	29.4 ± 6.3	0.088
Race, white, *n* (%)	88 (37.0%)	15 (44.1%)	73 (35.8%)	0.13
Comorbidities, *n* (%)				0.269
0	51 (21.4%)	4 (11.8%)	47 (23.0%)	
1	90 (37.8%)	16 (47.1%)	74 (36.3%)	
2+	97 (40.8%)	14 (41.2%)	83 (40.7%)	
Diabetes, *n* (%)	44 (18.5%)	4 (11.8%)	40 (19.6%)	0.275
COPD, *n* (%)	64 (26.9%)	10 (29.4%)	54 (26.5%)	0.72
Heart failure, *n* (%)	34 (14.3%)	6 (17.6%)	28 (13.7%)	0.596
Hypertension, *n* (%)	154 (64.7%)	22 (64.7%)	132 (64.7%)	>0.999
Osteoporosis, *n* (%)	45 (23.9%)	11 (40.7%)	34 (21.1%)	0.027
Prior fusion, *n* (%)	81 (34.0%)	22 (64.7%)	59 (28.9%)	<0.001
Type of malalignment, *n* (%)				0.004
Predominantly sagittal	83 (32.7%)	14 (41.2%)	69 (33.8%)	
Predominantly coronal	11 (4.3%)	0	11 (5.4%)	
Predominantly combined	47 (18.5%)	13 (38.2%)	34 (16.7%)	
Others	97 (38.2%)	7 (20.6%)	90 (44.1%)	
**Preoperative Radiographic Measurement**				
PT, mean ± SD°	25.3 ± 11.5	29.6 ± 11.6	24.6 ± 11.4	0.037
T1PA, mean ± SD°	25.2 ± 14.0	31.8 ± 12.7	24.0 ± 13.9	0.003
L1-S1 lordosis, mean ± SD°	−22.9 ± 32.9	−14.7 ± 28.4	−24.3 ± 33.4	0.039
L1-L4 lordosis, mean ± SD°	−30.6 ± 15.5	−25.4 ± 14.7	−31.6 ± 15.5	0.055
L4-S1 lordosis, mean ± SD°	−30.6 ± 15.5	−25.4 ± 14.7	−31.6 ± 15.5	0.042
CVA, mean ± SD (mm)	26.5 ± 27.1	32.8 ± 27.6	25.3 ± 26.9	0.166
PI, mean ± SD°	52.7 ± 15.8	54.2 ± 15.6	52.4 ± 15.9	0.470
SS, mean ± SD°	27.5 ± 13.4	24.7 ± 10.7	28.0 ± 13.7	0.116
cSVA, mean ± SD (mm)	30.5 ± 16.5	26.9 ± 16.9	31.1 ± 16.4	0.256
SVA, mean ± SD (mm)	71.7 ± 69.8	102.6 ± 51.9	66.4 ± 71.2	0.005

**Table 2:** Intraoperative variables.

Variables	Total Cohort = 238	Combined N = 34	Posterior Only N = 204	*p*-Value
**Intraoperative**				
Total instrumented levels, mean ± SD	10.5 ± 3.2	11.4 ± 3.3	10.4 ± 3.1	0.065
Presence of interbody, mean ± SD	70 (29.4%)	34 (100%)	36 (17.6%)	<0.001
Number of interbody, *n* (%)				<0.001
0	168 (70.6%)	0	168 (82.4%)	
1	44 (18.5%)	15 (44.1%)	29 (14.2%)	
2	18 (7.6%)	14 (41.2%)	4 (2.0%)	
3	5 (2.1%)	3 (8.8%)	2 (1.0%)	
4	3 (1.3%)	2 (5.9%)	1 (0.5%)	
Operative time (min), mean ± SD	419.9 ± 147.2	553.9 ± 177.4	397.4 ± 129.0	<0.001
EBL, mean ± SD	1452.6 ± 1232.6	1285.1 ± 1147.3	1480.7 ± 1246.8	0.194
Discharge disposition, *n* (%)				0.967
Home, *n* (%)	112 (50.7%)	17 (51.5%)	95 (50.5%)	
IPR, *n* (%)	71 (32.1%)	10 (30.3%)	61 (32.4%)	
SNF, *n* (%)	38 (17.2%)	6 (18.2%)	32 (17.0%)	
Length of stay (days), mean ± SD	7.2 ± 9.8	8.4 ± 10.7	7.0 ± 9.6	0.026

**Table 3:** Postoperative radiographic measurements and radiographic correction.

Variables	Total Cohort = 238	Combined N = 34	Posterior Only N = 204	*p*-Value
**Postop Radiographic Measurements**				
PT, mean ± SD°	24.5 ± 10.7	27.8 ± 12.3	23.9 ± 10.3	0.060
T1PA, mean ± SD°	22.3 ± 12.1	24.2 ± 13.4	22.0 ± 11.9	0.364
L1-S1 lordosis, mean ± SD°	−24.6 ± 35.9	−28.3 ± 35.8	−23.9 ± 35.9	0.527
L4-S1 lordosis, mean ± SD°	−28.2 ± 12.6	−28.6 ± 15.9	−28.1 ± 11.9	0.853
CVA, mean ± SD (mm)	21.4 ± 21.3	24.5 ± 23.4	20.9 ± 21.0	0.459
PI, mean ± SD°	52.4 ± 14.1	56.4 ± 13.3	51.6 ± 14.2	0.115
SS, mean ± SD°	27.2 ± 10.0	29.3 ± 9.2	26.8 ± 10.1	0.202
cSVA, mean ± SD (mm)	31.3 ± 15.6	31.1 ± 14.6	31.4 ± 15.8	0.929
SVA, mean ± SD (mm)	50.5 ± 58.2	46.1 ± 50.6	51.3 ± 59.6	0.658
**Postop Radiographic Correction**				
PT, mean ± SD°	7.6 ± 7.2	8.7 ± 7.8	7.4 ± 7.1	0.217
T1PA, mean ± SD°	10.1 ± 8.7	13.4 ± 8.7	9.5 ± 8.6	0.005
L1-S1 lordosis, mean ± SD°	−5.0 ± 21.4	−14.3 ± 25.6	−3.2 ± 20.2	<0.001
L4-S1 lordosis, mean ± SD°	1.8 ± 14.5	−4.7 ± 16.4	3.2 ± 13.7	0.008
CVA, mean ± SD (mm)	31.4 ± 32.3	39.7 ± 39.3	29.9 ± 30.8	0.295
PI, mean ± SD°	10.6 ± 10.9	11.4 ± 11.7	10.4 ± 10.8	0.609
SS, mean ± SD°	8.5 ± 7.7	8.5 ± 7.9	8.5 ± 6.5	0.966
cSVA, mean ± SD (mm)	48.0 ± 47.7	65.3 ± 44.8	44.8 ± 47.7	0.007
SVA, mean ± SD (mm)	48.0 ± 47.7	65.3 ± 44.7	44.7 ± 47.6	0.005

**Table 4:** Multivariable linear regression, controlling for age, BMI, and previous fusion.

		Univariate		Multivariable	
Outcome	Independent Variable	β (95%CI)	*p*-Value	β (95%CI)	*p*-Value
T1PA correction	AP vs. P	3.94 (0.56–7.3)	0.022	3.72 (0.14–7.31)	0.041
L1-S1 correction		−11.1 (−19.2, −2.9)	0.008	−11.18 (−19.71, −2.65)	0.010
L4-S1 correction		−8.0 (−13.9, −2.1)	0.008	−7.26 (−13.65, −0.88)	0.026
SVA correction		20.5 (1.65–39.4)	0.033	20.49 (0.71–40.28)	0.042

**Table 5:** The impact of surgical approach on mechanical complications and patient-reported outcome measures.

*p*				
Variables	Total Cohort = 238	Combined N = 34	Posterior Only N = 204	*p*-Value
**Postoperative Outcomes**				
Mechanical complication	146 (61.3%)	22 (64.7%)	124 (60.8%)	0.664
Radiographic PJK	110 (47.8%)	18 (54.5%)	92 (46.7%)	0.404
Pseudarthrosis	66 (27.7%)	7 (20.6%)	59 (28.9%)	0.315
RF	46 (19.3%)	5 (14.7%)	41 (20.1%)	0.461
DJK	8 (3.4%)	1 (2.9%)	7 (3.4%)	>0.999
MCID ODI *	61 (54.0%)	5 (38.5%)	56 (56.0%)	0.233
MCID NRS Back *	58 (51.3%)	5 (38.5%)	53 (53.0%)	0.324
MCID NRS Leg *	67 (65.7%)	7 (53.8%)	60 (67.4%)	0.336
MCID EQ-5D *	67 (65.7%)	7 (53.8%)	60 (67.4%)	0.323

* missing values.

## Data Availability

For data inquiries, please contact Scott L. Zuckerman.
